# Diagnostic Methods of Atherosclerotic Plaque and the Assessment of Its Prognostic Significance—A Narrative Review

**DOI:** 10.3390/jcdd11110343

**Published:** 2024-10-30

**Authors:** Paweł Gać, Anna Jakubowska-Martyniuk, Aleksandra Żórawik, Wojciech Hajdusianek, Dawid Żytkowski, Tomasz Matys, Rafał Poręba

**Affiliations:** 1 Department of Environmental Health, Occupational Medicine and Epidemiology, Wroclaw Medical University, Mikulicza-Radeckiego 7, 50-368 Wroclaw, Poland; 2 Centre of Diagnostic Imaging, 4th Military Hospital, Rudolfa Weigla 5, 50-981 Wrocław, Poland; 3 Department of Angiology and Internal Diseases, Wroclaw Medical University, Borowska 213, 50-556 Wroclaw, Poland

**Keywords:** cardiovascular, atherosclerosis, diagnostics, plaque, prognosis

## Abstract

Cardiovascular diseases (CVD) are a leading cause of death. The most notable cause of CVD is an atherosclerotic plaque. The aim of this review is to provide an overview of different diagnostic methods for atherosclerotic plaque relevant to the assessment of cardiovascular risk. The methods can be divided into invasive and non-invasive. This review focuses on non-invasive with attention paid to ultrasonography, contrast-enhanced ultrasonography, intravascular ultrasonography, and assessment of intima-media complex, coronary computed tomography angiography, and magnetic resonance. In the review, we discuss a number of Artificial Intelligence technologies that support plaque imaging.

## 1. Introduction

Cardiovascular diseases are one of the leading causes of death in the European Union with ischemic heart disease and stroke responsible for the majority of deaths from cardiovascular disease. However, it should be emphasized that age-standardized death rates of cardiovascular diseases have decreased recently by approximately 10%. [1,2]. Given the seriousness of this problem, a great deal of scientific effort has been devoted to addressing this issue, resulting in the development of many guidelines, such as the European Society of Cardiology Guidelines. Of these, particular attention to the problem of atherosclerotic plaque was given in the 2021 ESC Guidelines on cardiovascular disease prevention in clinical practice [3]. The guidelines emphasize the importance of atherosclerotic cardiovascular disease and its risk factors such as, inter alia, low-density lipoprotein cholesterol, blood pressure, cigarette smoking, or diabetes mellitus leading to atherosclerotic plaque formation that leads to vascular occlusion. Atherosclerosis begins with the disruption of the most inner layer of the vascular wall, which is an endothelium, which is mainly caused by the exposition to cardiovascular risk factors. The plaque may be initially asymptomatic, and, in some patients, it may even remain so throughout their life; however, especially in patients with additional risk factors, it may lead to further complications [3,4].

Diagnostic imaging, however, participates not only in evaluation of risk in the area of cardiological prevention, but assists in diagnosing patients suspected of acute coronary syndromes. In particular, coronary computed tomography angiography (CCTA) can help to assess patients with no ECG changes and uncertain high-sensitivity cardiac troponin [5]. Similarly, for patients with suspected chronic coronary syndrome with an appropriate pre-test likelihood assessment of disease, CCTA is a recommended diagnostic procedure [6].

There are studies reporting that plaque can excessively enlarge in the months before clinical incident. In a study conducted by Hackett et al. it was based on the records of all their patients who happened to have had coronary arteriography performed during a clinically stable phase of their disease before and after AMI [7]. Therefore, appropriate imaging is particularly important [8,9].

**Figure 1:**
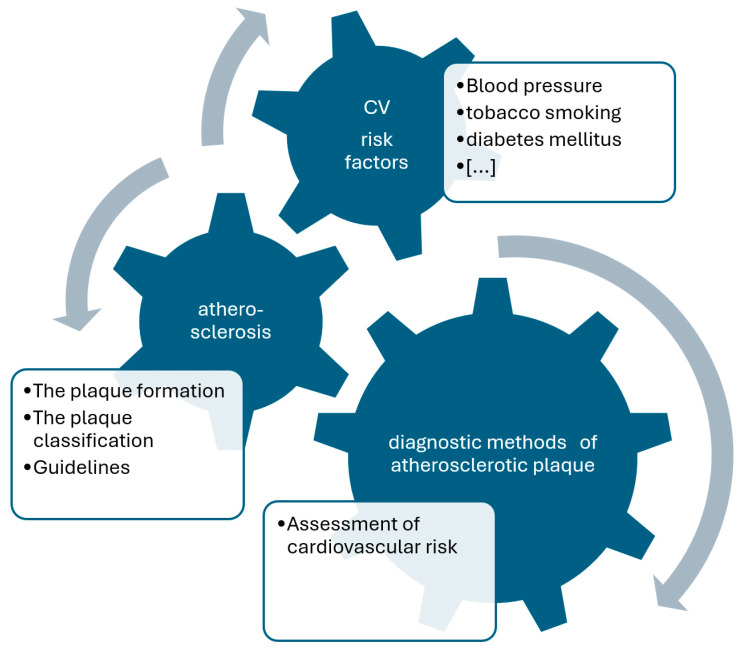
Methodological approach of the current review. CV—cardiovascular.

The plaques have been histologically classified by Stary et al. based on their components [9,10]. Such components include, for instance, atherogenic lipoprotein, macrophage foam cells, lipid-laden smooth muscle cells, extracellular lipid droplets or calcium. It should be emphasized that some plaque poses a greater threat than others—particularly type IV and V. There is also a noticeable variation in the pattern of plaque growth. Plaque of types I–IV grow mainly due to lipid accumulation, whereas type V is caused by smooth muscle and collagen increase and type VI is caused by thrombosis or hematoma [10]. The main methodological concepts of current review are presented in Figure 1.

The aim of this review is to provide an overview of different radiological diagnostic methods: ultrasonography, computed tomography, magnetic resonance imaging, positron emission tomography of atherosclerotic plaque which are relevant to the assessment of cardiovascular risk.

## 2. Imaging of Atherosclerotic Plaque Morphology

**Figure 2:**
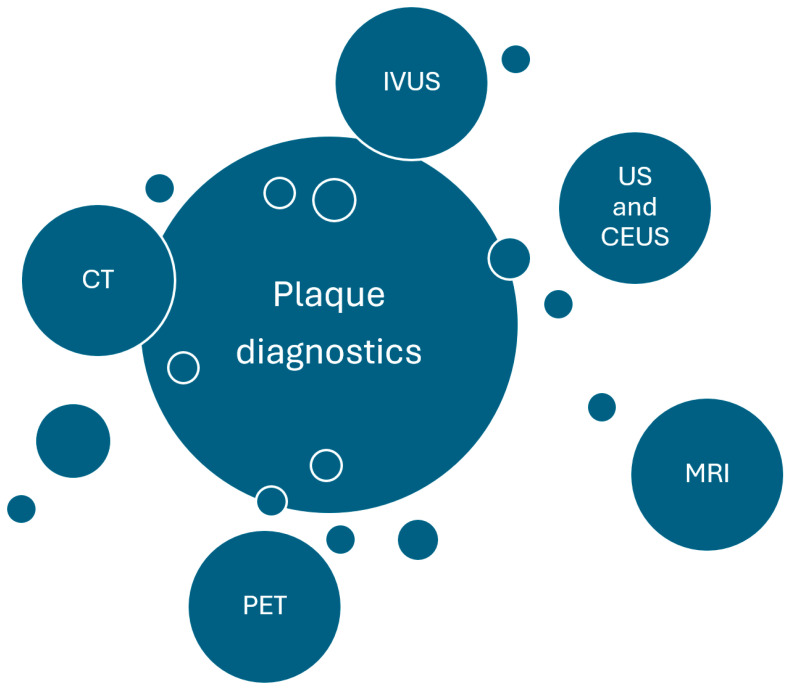
Main research concepts. CT—computed tomography, IVUS—intravascular ultrasound, CEUS—contrast—enhanced ultrasound, MRI—magnetic resonance imaging, PET—positron emission tomography, US—ultrasound.

Currently, many invasive and non-invasive imaging methods are used to study atherosclerosis; most specify lumen diameter or stenosis, wall thickness, and plaque volume [11]. A wide variety of techniques are used in clinical practice. They include ultrasound, computer tomography (CT), magnetic resonance tomography (MRI), positron emission tomography (PET), single-photon emission computed tomography (SPECT), and photon-counting detector CT (PCD-CT) [12,13]. The imaging of plaque is also the subject of guidelines of American Society of Neuroradiology, which emphasizes that not only the measurements of luminal stenosis, but also advanced wall imaging is important in identifying plaque that poses a greater risk. The main research concepts are presented in Figure 2.

### 2.1. Ultrasound Imaging Techniques

**Figure 3:**
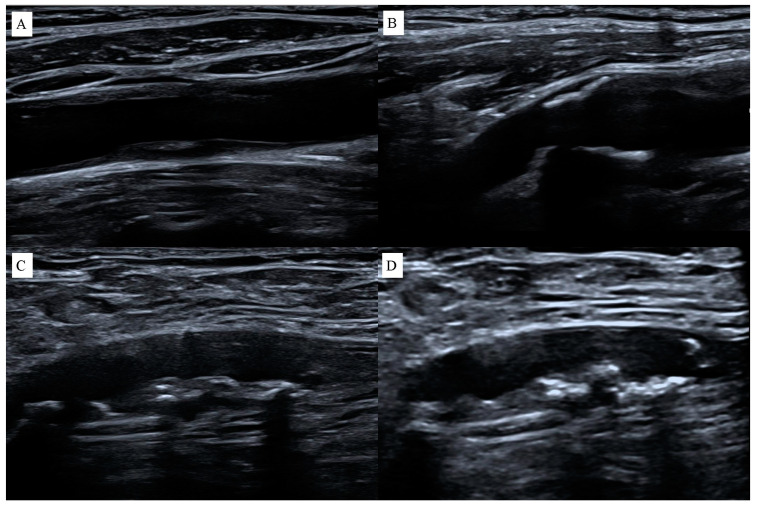
Atherosclerotic plaques on ultrasound examination: (**A**) heterogeneous plaque in the common carotid artery, (**B**) homogeneous hyperechoic plaques in the carotid bulb, (**C**) heterogeneous plaque in the superficial femoral artery, (**D**) hyperechoic plaque in the superficial femoral artery.

Ultrasound imaging techniques (USG) help to find vulnerable atherosclerotic plaques [12]. This method is based on transmitting and receiving high-frequency sound waves [11]. This provides the high spatial resolution required for measuring intima-media thickness (IMT). However, higher frequency is also limited by the depth of body penetration [14]. The time between transmission and reception of a wave is related to the distance between the source and the reflector [11]. Due to signal attenuation problems, non-invasive ultrasound for imaging blood vessel wall is generally limited to shallow vascular beds such as carotid, femoral, and other peripheral arteries. Atherosclerotic plaque can be directly visualized on B-mode ultrasound, intravascular ultrasound (IVUS), and three-dimensional (3D) ultrasound [15]. The thickness of the artery wall and the structure and composition of atherosclerotic plaque can be measured [14]. The echogenicity of the plaque reflects its characteristics. Hypoechoic heterogeneous plaque is associated with both intraplaque hemorrhage and lipids, whereas hyperechoic homogeneous plaque is mainly fibrous [11], Figure 3.

**Figure 4:**
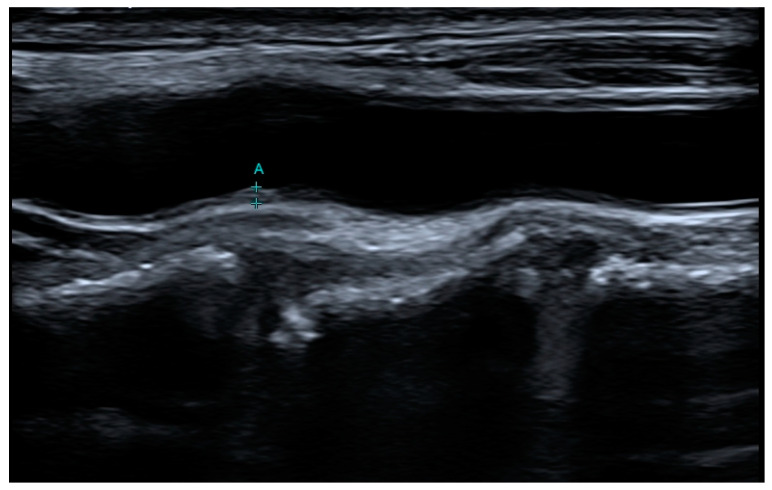
Example of measurement of intima media thickness (IMT) in ultrasound examination of the carotid arteries. A—IMT measurement, + measurement markers.

B-mode ultrasound of the carotid arteries can identify plaques and measure intima-media thickness (IMT) [16]. Abnormal thickening of the carotid IMT is thought to be a marker of generalized atherosclerotic disease. However, different sources provide nonidentical limit values for IMT. Ibanez et al. say that normal IMT has been determined to be approximately 0.5 to 1.1 mm, values > 1.1 mm may indicate the presence of atherosclerotic plaque [14]. However, the Mannheim consensus suggests at least 0.5 mm or 50% of the surrounding IMT value and thickness of at least 1.5 mm [17]. On the other hand, Chuan-Wei Yang et al. assume presence of the intima–media’s focal thickening > 1 mm that bulges out into the carotid artery’s lumen with at least twice the thickness of the IMT on either side [18]. And, finally, the American Society of Echocardiography defines plaque as any thickening of atherosclerotic origin that intrudes into the lumen of carotid artery, or an IMT of at least 1.5 mm [19]. Abnormal thickening of the carotid IMT is thought to be a marker of generalized atherosclerotic disease. Normal IMT has been determined to be approximately 0.5 to 1.1 mm, with values > 1.1 mm indicating the presence of atherosclerotic plaque. The use of this index as a vascular marker is based partly on the assumption that carotid IMT > 75th percentile for age indicates generalized atherosclerosis [14]. Although ultrasonography has the advantage of being non-invasive and enabling qualitative assessment of carotid plaques, image quality is limited by echo windows and calcification [16]. IMT measurement has proved to be a useful research technique when quality can be rigorously controlled and many patients are involved; however, it is less useful in a clinical setting for monitoring an individual patient [14]. An example IMT measurement is shown in Figure 4.

Due to limitations caused by the physics of ultrasound examinations, the examination is dependable only at the far arterial wall and does not indicate whether the thickening is because of intima or media infiltration or hypertrophy. As with other USG methods, this technique is operator-dependent and has lower reproducibility [11].

To indirectly find out if a patient has blood flow problems (for example due to stenosis caused by atherosclerotic plaque), other tests might be chosen, like a stress echocardiography. This test uses intravenous vasodilators such as adenosine or dobutamine. There are different stresses of similar diagnostic and prognostic accuracy. Among them dobutamine is the best for viability. The choice of one test over the other depends on patient characteristics, local drug costs, and the physician’s preference. Stress echocardiography is a good choice due to it lower cost, wider availability and for the radiation-free nature [20,21].

### 2.2. The Use of Contrast in USG Imaging of Atherosclerosis

Another method is to inject a contrast agent into liposomes [12]. Contrast-enhanced ultrasound (CEUS) can provide information about plaque composition, and structural information [22]. The quantitative assessment of microbubble retention in the carotid plaque on CEUS is a technique that has promise as a tissue-specific marker of inflammation and a potential role in risk stratification of atherosclerotic carotid stenosis [23]. CEUS enables an assessment of myocardial perfusion, a function of left ventricle and intracardiac thrombus and endocardial borders [24]. Microbubbles are retained in inflamed tissue, it is possible that CEUS could be translated into clinical practice, where it may have a role in monitoring therapy or selecting patients for surgical procedures [22]. There are available USG devices that have preprogrammed settings for CEUS. To avoid destruction of the microbubbles, you select a low mechanical index, which allows continuous image acquisition (0.1–0.3) or middle-high (0.3–0.5) mechanical index, which requires intermittent imaging allowing the replenishment of destructed microbubbles [24]. UCAs (Ultrasound Contrast Agents) are administered safely in various applications [25] with a very low rate of adverse reactions (about 0.014%) [26,27]. Conducting a laboratory assessment of the liver, thyroid, or kidney function before administration is not required [28]. Contraindications for the contrast agent administration are allergy to the agent, large right to left shunt, and an unstable condition [24]. The overall reported rate of fatalities attributed to one UCA, SonoVue™ (Bracco, Milan), is low (14/2,447,083 exposed patients; 0.0006%) and compares favorably with the risk of fatal events reported for iodinated contrast agents (approximately 0.001%) [25]. The limitations of this method is that CEUS is significantly dependent on operator skill, the cost of contrast media is not negligible, and the image lacks a wide scope and therefore has difficulty exploring some deep regions [27].

The novel imaging technique is a 3-dimensional vascular ultrasound (3DVUS) [29]. There are reports that say that 3DVUS is a more comprehensive evaluation of overall atherosclerosis burden, which avoids the drawbacks of 2DVUS, and offers reproducibility of plaque measurements. The volumetric-linear probe uses the “mechanical-sweep” method and enables accurate measurements of atherosclerosis from early to more advanced disease stages regardless of plaque size [30].

Although 3DVUS allows for the detection and assessment of atherosclerotic plaques in arteries, such as the femoral or carotid artery, it is not accurate in measuring them in deeper vessels such as the aorta [30]. It is inexpensive and radiation-free and has the potential to become an important screening device for identifying patients in high-risk groups [29].

### 2.3. Intravascular Ultrasonography Assessment

Intravascular ultrasound (IVUS) is an innovative approach to arterial wall imaging, enabling direct real-time imaging of atherosclerosis and providing a cross-sectional, tomographic perspective of the vessel and atherosclerotic disease [11]. It is a catheter-based test, which, in addition to determining the size of the coronary lumen, allows for obtaining an image of the thickness and acoustic density of the entire vessel wall [14]. It is regulated by accurate and deeply penetrating imaging capabilities with a distributed signal converted in real time into a two-dimensional (2D) video image. Grayscale IVUS enabled the in vivo assessment of vessel wall dimensions, phenotypic features, distribution, and severity of atherosclerotic lesions [31]. The advantage of IVUS over regular US techniques is that it can provide data on the structure of atherosclerotic plaque. The liposome has a layered structure, which allows it to capture gas bubbles that can effectively reflect sound waves and produce acoustically reflective liposomes [12]. Liposomes can be conjugated to antibodies such as anti-fibrinogen or anti-ICAM-1 to enhance platelet recognition and targeting [12]. The current generation of catheters (incorporating a transducer) have a diameter of 0.96 to 1.17 mm and provide high image quality. Based on echogenicity, atherosclerotic plaque can be divided into three categories: (1) highly echogenic areas with acoustic shadows, often corresponding to calcified tissue; (2) hyperechoic areas indicating fibrosis or microcalcifications; or (3) hypoechoic areas consistent with thrombotic or lipid-rich tissue or a mixture of these [11].

Subsequent advances in IVUS processing, and, in particular, the analysis of the radiofrequency ultrasonic backscatter signal (IVUS-RF), also known as virtual histology intravascular ultrasound (VH-IVUS), allowed a real-time cross-sectional and longitudinal three-dimensional (3D) visualization of a vessel that broadened the knowledge on the composition and mechanical properties of the vulnerable plaque [31]. VH-IVUS can precisely detect the presence of fibrous, fibro-lipid, calcified, and necrotic areas in plaques [22]. The predictive accuracy of in vivo IVUS-VH can be degraded by the presence of intramural thrombus [15].

IVUS may be useful in selecting the most appropriate option of transcatheter therapy (rotational atherectomy, stents, etc.)—lesions with calcification would be expected to be more rigid and, therefore, prone to rupture in response to the mechanical stress of balloon dilation, whereas softer, lipid-rich, noncalcified plaque may stretch but not fracture [11]. Studies that have compared ultrasound measurements with histological findings have shown that the IMT of posterior (far) wall IMT of the carotid artery as measured with the use of US reflects the true thickness of the wall, although measurements recorded with US may be slightly different than estimates attained by histology. Values obtained by measuring the anterior (proximal) wall of the carotid artery are less accurate [14]. Based on research conducted by Gernot Schulte-Altedorneburg et al., it was noticed that values obtained by ultrasound always turned out to be smaller than those obtained histologically, indicating a systematic discrepancy [32]. One of USG’s advantages is its great spatial resolution, due to its high frequency (up to 50 MHz). On the other hand, IVUS is an invasive procedure [33].

### 2.4. Computed Tomography Assessment

**Figure 5:**
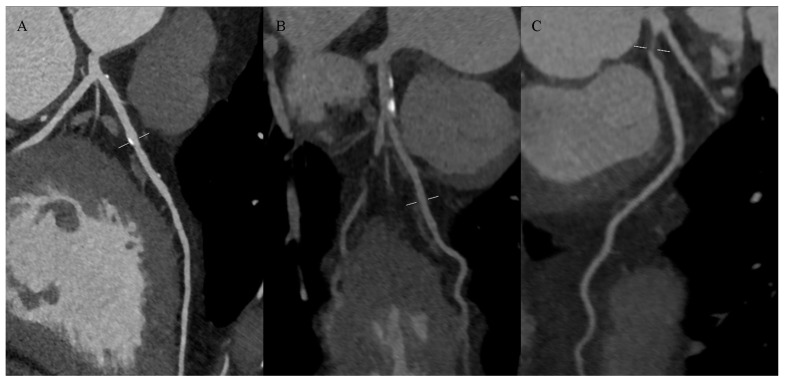
Atherosclerotic plaques on coronary computed tomography angiography: (**A**) calcified plaques in the left anterior descending artery (LAD), (**B**) mixed plaques in the LAD, (**C**) non-calcified concentric plaque in the LAD.

Computed tomography (CT) is fast and relatively inexpensive. With a bolus injection of a contrast agent, CT is suitable for detecting calcifications in atherosclerotic plaque and fibrous tissue [12]. However, the lipid-rich necrotic core could only be adequately quantified in certain subsets of plaque, and hemorrhage and thrombus could not reliably be distinguished from lipids. Plaque density measured in Hounsfield units showed significant overlap between densities associated with lipid-rich necrotic core, connective tissue, and hemorrhage [15]. This method is not effective in detecting other components of high-risk plaque: thin-capped fibroma and the presence of inflammatory cells [16]. Examples of hyperdense (calcified), mixed-dense, and hypodense (noncalcified) atherosclerotic plaques on CTA of coronary arteries are presented in Figure 5.

**Figure 6:**
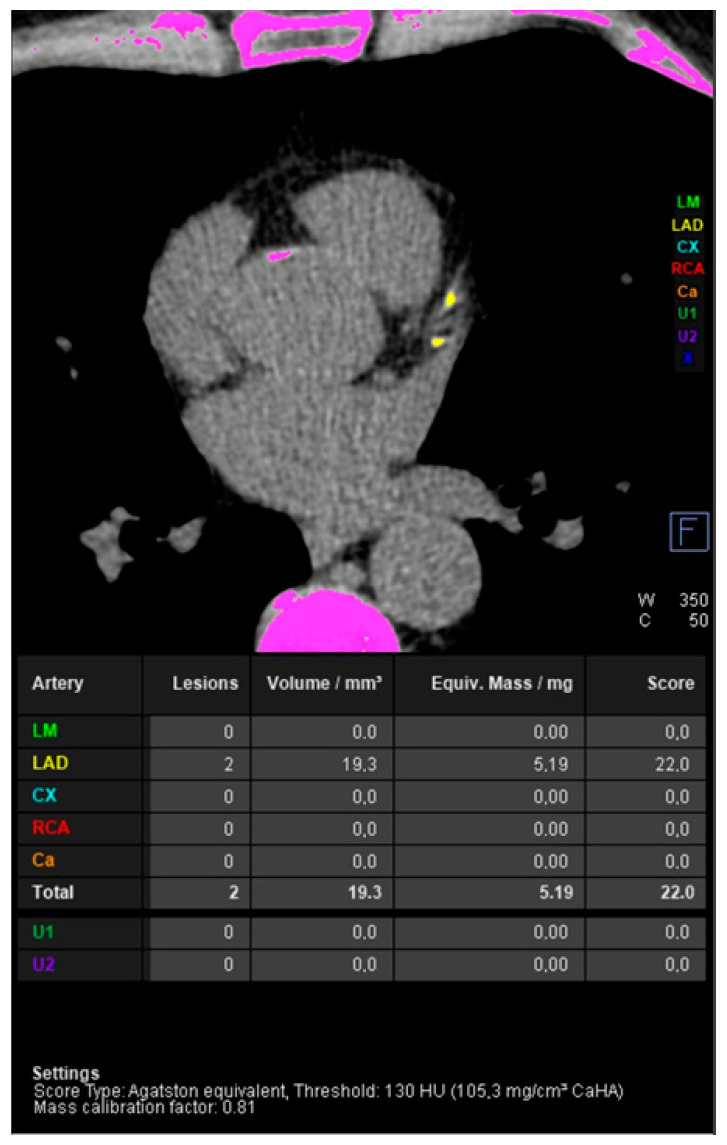
Coronary artery calcium score measurement using non-contrast computed tomography. Light green indicates calcifications in the left main (LM), yellow in the left anterior descending (LAD), blue in the left circumflex (CX), red in the right coronary artery (RCA), orange in other coronary branches (Ca), dark green and purple in extracoronary structures (U1 and U2). The application indicates voxels proposed as meeting the calcification criterion in pink.

Computed tomography uses two techniques to image atherosclerosis: one is the more traditional angiographic technique (CTA), which allows the assessment of narrowing of the lumen of the artery but requires the use of a contrast agent. Another technique is direct calcium visualization and related calcium quantification methods such as calcium scoring [15]. An example of coronary artery calcium score measurement using CT is shown in Figure 6.

It has been shown that the amount of calcium detected in coronary vessels correlates with the extent of coronary atherosclerosis detected histologically [22,34]. This allows more accurate assessment of coronary plaque burden. Assesses presence of both obstructive and non-obstructive disease and analysis of plaque composition [35]. The second category includes: electron-beam CT (EBCT), multiple-row detector CT (MDCT/MSCT) and dual-source tomography (DSCT). EBCT uses stationary tungsten rings to generate X-ray images at 3 mm slice thickness from which a coronary artery calcium score is calculated to assess cardiovascular risk. In contrast, the latter uses a continuously rotating X-ray source to obtain 0.5–0.75 mm slices during a single patient breath hold [22]. Dual source computed tomography (DSCT) is used in the assessment of atherosclerotic plaque by simultaneously capturing images from two X-ray systems, which can achieve increased temporal resolution and acquisition speed combined with significantly reduced radiation dose [16]. Nevertheless, this technique cannot be used to differentiate thin-cap fibroatheroma, only to assess the features of calcifications and fibro-fatty tissue in the coronary plaque [36]. Novel photon-counting detector CT (PCD-CT) has the potential to address the limitations of previous CT systems, such as insufficient spatial resolution, limited accuracy in detecting small low-contrast structures, or missing routine availability of spectral information [13]. The photon-counting computed tomography (PCCT) has a significant advantage in the imaging of coronary arteries and enables a wider examination of the plaque structure [37].

### 2.5. Optical Coherence Tomography

Recent studies have shown that optical coherence tomography (OCT) is an accurate method for assessing the thickness of the fibrous cap in atherosclerotic plaques [12], enabling the identification of thin caps and plaque ruptures and erosion [35]. OCT uses near-infrared light emitted through a fiberoptic wire with a rotating lens to achieve an exceptionally high spatial resolution (10–15 μm), providing accurate measurement of fibrous cap thickness with strong correlation to histology, and good sensitivity and specificity to distinguish plaque type [38]. OCT has proven useful in assessing intraplaque neovascularization, which is a key factor contributing to atherosclerotic plaque growth and instability [31]. Unfortunately, the distinction between calcium and lipids in plaques can be difficult with OCT due to limited tissue penetration (up to 3 mm), which makes it difficult to estimate the entire plaque volume [12]. Moreover, for image acquisition, a blood-free field is needed, which can be achieved through the supply of saline or contrast flushing during pullback [38]. OCT has been found to be useful for assessing developmental processes, including thrombus formation and calcifications important for atherosclerotic plaque progression [31,39].

### 2.6. Magnetic Resonance Imaging

Other commonly used methods include magnetic resonance imaging (MRI) and magnetic resonance angiography (MRA), which use gadolinium and iron oxide derivatives as contrast enhancement with a resolution of 10–100 microns to visualize the structure of atherosclerotic lesions [12]. In addition to the traditional contrast, MRI offers the ability to probe atherosclerotic plaque for diffusion, contrast uptake, dynamic contrast permeability, magnetization transfer, and others [15]. MR provides imaging without ionizing radiation and can be repeated sequentially over time [11]. Early studies have shown that the surface area of atherosclerotic plaque tissue components with a lipid-rich core assessed by MRI correlates with a histopathological assessment [16]. In clinical practice, MRI mainly visualizes signals from protons in free water, triglycerides, and free fatty acids [22], differentiates plaque components based on biophysical and biochemical parameters, such as chemical composition and concentration, water content, physical state, and molecular movement [11]. Macromolecules, for example proteins or cholesterol crystals, are not involved in conventional MR signals due to a noticeably short T2 signal [16,40]. MRI enables not only the quantitative assessment of the size of the atherosclerotic plaque, but also the assessment of intra-plaque hemorrhage and the integrity of the fibrous sheath [14], and it provides the ability to distinguish the vessel lumen from the vessel wall [41]. Non-contrast T1-weighted magnetic resonance imaging can identify the presence of high-risk plaques and thrombi [31], which uses a high T1 signal associated with methemoglobin, a key component of fresh thrombus [35]. Additionally, it can detect positive arterial remodeling in asymptomatic patients with subclinical atherosclerosis [31].

Coronary magnetic resonance imaging (MRI) is a rapidly developing method that, thanks to recent technological improvements, can provide reliable imaging of the proximal and middle vessels [38]. However, this technique is not optimal for quantifying lumen area/volume because it is prone to unwanted signal loss due to complex flow patterns [15]. When using “bright blood” contrast-free techniques for coronary MRA, which relies on a high T2/T1 ratio of blood to function as an internal contrast agent, there is a need to potentially avoid nephrotoxic contrast agents [38].

### 2.7. Positron Emission Tomography

Methods such as positron emission tomography (PET) and single photon emission computed tomography (SPECT) are gaining popularity because they use imaging elements such as 18F, 64Cu, 11C/99mTc, 123/124/125/131I, 111In tracers [14]. Radioisotope decay is detected in order to produce the signal, measured as standardized uptake values or tissue-to-background ratios [22]. The efficiency of PET is much greater, and the technique provides higher resolution, less noise, and less radiation exposure than SPECT [14]. Additionally, a prevalence of PET over other techniques, including SPECT and MRI, is its greater sensitivity in detecting molecular signals, but limited spatial resolution means that images must be co-registered (like SPECT) with CT or MR to accurately localize the anatomical signal PET [38]. The development of hybrid PET/CT scanners with improved imaging allowed the assessment of the activity of atherosclerotic disease [35]. PET is used to detect cellular activity and assess biological processes relevant to atherosclerosis, such as arterial inflammation, hypoxia, neo-angiogenesis, and microcalcification [31].

The PET study uses 18F-fluorodeoxyglucose (FDG), a commonly used radiolabeled glucose analog for various diagnostic purposes [38], which accumulates in proportion to metabolic activity [14] and is captured by macrophages [31]. It has been shown that numerous macrophages reside in ruptured plaques [12]. 18F-FDG accumulates in the arterial wall in direct proportion to the degree of cellular glycolysis, respectively, reflecting the density of atherosclerotic plaque macrophages and the degree of inflammation [31], as a non-specific marker [38]; therefore, it is worth noting that FDG uptake may have added value in detecting the condition inflammation (e.g., in psoriasis, RA or HIV) [22] or diabetes [35]. High uptake of 18F-FDG by myocardial cells often prevents the interpretation of coronary signals [38], but uptake can be reduced by preparation before the test with a low-carbohydrate, high-fat diet [23]. Importantly, FDG/PET-CT has been shown to be highly reproducible in assessing the degree of FDG uptake by the vessel wall [14]. FDG uptake can be decreased by medication. This may lead to adapting it as an endpoint in various trials which target the anti-inflammatory effects of different therapies [35].

Another PET tracer used for the dynamic assessment of microcalcifications in coronary vessels is 18F-sodium fluoride (18F-NaF), which is commonly used as a marker of bone mineralization in skeletal imaging [12]. In this case, uptake by the myocardium has no effect on the signal [31]. In aortic stenosis, areas of increased 18F fluoride activity predict where new macroscopic calcium deposits will be deposited, providing excellent prediction of progression in the valve calcification score [35].

The use of other tracers is also being studied, such as 68Ga-DOTATATE [31], 18F-fluorocholine (18F-FCH) or 11C-PK11195 [35]. There was decreased background heart cell uptake with tracers when compared with 18F-FDG. Therefore, they are preferable for coronary artery imaging [31].

Radioactive isotopes used to produce SPECT tracers typically have longer half-lives and are more widely available than those used in PET [16]. Additionally, SPECT is widely available (and cheaper) than PET, but is susceptible to artifacts, especially those caused by motion and soft tissue, and requires significant radiation exposure [42].

The table for comparison of the different imaging modalities is presented below as Table 1.

### 2.8. Multimodality Imaging

Multimodality consists of combining two or more techniques [43]. One of the techniques consists of near-infrared spectroscopy (NIRS) and intravascular ultrasound (IVUS), in which NIRS is responsible for the assessment of plaque with high lipid content and IVUS is responsible for the dimension of plaque measurements [44]. This technique and other similar techniques are interesting aspects of multimodality, which is a development of dual-probe catheters for an invasive plaque assessment. Another available example is the combination of IVUS and optical coherence tomography [45]. Another example of multimodality is the combination of magnetic resonance imaging and positron emission tomography to enrich the examination with inflammation analysis due to the accumulation of radionuclide in macrophages [46].

## 3. Artificial Intelligence and Atherosclerotic Plaque Imaging

### 3.1. Artificial Intelligence in Atherosclerosis Assessment

Nowadays, there are attempts to increase the share of artificial intelligence (AI) in everyday diagnostics, including the detection of asymptomatic atherosclerosis. AI is playing an increasingly significant role in supporting image processing and interpretation, offering greater efficiency, fewer human errors, and better diagnostic accuracy, without increasing costs and workload [41], enabling accurate measurement of atherosclerotic plaque volume and stenosis severity based on CCTA scans [47]. Combining human knowledge with artificial intelligence can facilitate the reliable and accurate interpretation of images obtained using CT, MR, PET, intravascular ultrasound, and OCT [48]. One of the many applications of artificial intelligence is the creation of predictive models by exposure to substantial amounts of data in order to match or exceed the capabilities of simple visual assessment or manual measurement [47]. The use of AI, which is not guided by any generally accepted assumptions, allows the exploration of all available data for non-linear patterns that can predict the risk of a specific person, i.e., precise risk stratification [49]. At the same time, rapid improvements in artificial intelligence algorithms will facilitate full automation of software-based plaque quantification [50], an evolving field with the potential to have a profound impact on clinical practice [47].

### 3.2. Legal Aspects of Using Artificial Intelligence in Radiology

Artificial Intelligence (AI) in radiology represents one of the most innovative applications of technology in medicine. It enables the automatic analysis of medical images, assisting doctors in diagnosing diseases, monitoring treatment progress, and planning therapy. Despite numerous benefits, the implementation of AI in this field involves significant legal challenges.

Ensuring patient safety is a fundamental aspect of introducing AI into radiology. Legal regulations concerning medical devices, such as the European Parliament and Council Regulation (EU) 2017/745 on medical devices (MDR), require these devices to undergo rigorous conformity assessments before being placed on the market [51]. AI algorithms must be thoroughly evaluated for efficacy and safety, and their results must be transparent and accessible to regulatory bodies.

Processing medical data using AI in radiology imposes high requirements for data protection. According to the General Data Protection Regulation (GDPR), medical data are classified as a special category of data that requires additional protection [52]. Therefore, the use of AI for analyzing medical images must comply with principles of data minimization, purpose limitation, and data integrity and confidentiality. It is also necessary to obtain patient consent for processing their data unless there is another legal basis.

One of the biggest legal challenges related to AI in radiology is the issue of liability for diagnostic errors. Traditionally, the responsibility for diagnostic errors lies with the physician, but the use of AI complicates the situation. Three main scenarios of liability can be distinguished: the liability of the software manufacturer, the liability of the user (physician), and shared liability. In practice, resolving liability issues may require analyzing the specific circumstances of a given case, including whether the algorithm operated as intended and whether the user properly interpreted its results [53].

The dynamic development of AI in radiology requires flexible and adaptive legal regulations. Current legal frameworks may not keep pace with the rate of innovation, necessitating continuous updates. In particular, there is a need to develop specific regulations concerning the certification of AI algorithms, real-time monitoring of their performance, and ensuring transparency in AI decision-making processes. It is also important for these regulations to be harmonized at the international level to ensure consistency and facilitate data exchange and cross-border cooperation [54].

The application of artificial intelligence in radiology holds immense potential but also involves significant legal challenges. Key issues include ensuring patient safety, protecting personal data, and determining legal liability. Future legal regulations must be flexible and adapted to the dynamically evolving technology to effectively support its safe and efficient use in medical practice.

## 4. Discussion

Despite great progress and effort in reducing atherosclerotic plaque formation as part of the treatment of patients after acute coronary syndromes and in secondary prevention, the incidence of atherosclerosis is exceedingly high. The assessment of plaque stability remains an important prognostic factor, so the development of diagnostic methods remains an important therapeutic issue. Atherosclerosis appears to be an irreversible process, although studies to date report possible plaque regression with intensive drug therapy [55,56,57,58,59]. Atherosclerosis is a major contributor to cardiovascular disease, which is the most common cause of death worldwide [58]. This group of diseases includes stroke, which is one of the most common causes of death. Annually, there are about twelve million cases of stroke worldwide, of which about 62% are ischemic in origin [58,59]. About 87% of ischemic stroke cases are associated with the presence of modifiable risk factors, such as lipid levels, as one of the most common causes of stroke is atherosclerotic disease, usually affecting the proximal portion of the internal carotid arteries [59]. This is why early diagnosis and treatment of pathological conditions that can easily improve the prevention of atherosclerosis is so important.

Undoubtedly, an unprecedented achievement is the development of methods in the field of intravascular imaging diagnostics represent a major advance in terms of assessing the composition and morphology of atherosclerotic plaque [57,60]. Previous studies have reported that accurate diagnosis of plaque rupture, plaque erosion, or calcified nodule (the three most common causes for the onset of coronary thrombosis [61]) can be helpful in choosing appropriate therapy specific to unstable lesion types in acute coronary syndromes [60,62,63]. Coronary thrombosis based on plaque rupture more often results in no-flow and distal embolization after percutaneous coronary intervention and larger myocardial infarct size [64]. Acute coronary syndromes occurring from coronary thrombosis based on plaque erosion have a better clinical prognosis compared to those with plaque rupture and, in addition, are potentially stabilizable without stent implantation, only with anticoagulant treatment [63,65]. In the studies described so far, acute coronary syndromes due to calcified nodules have been associated with incidents of incomplete stent expansion, resulting in an increased risk of restenosis and stent thrombosis [66].

With the development of novel imaging methods for atherosclerotic plaque, the expected prognostically significant endpoints can be identified with increasing precision. This now makes it possible not only to assess the burden of atherosclerosis, but also to accurately determine the composition of the plaque. With these advances, the effectiveness of the anti-atherosclerotic treatment used can be accurately assessed and the progression of the disease can be controlled. The need for further development of these imaging techniques is dictated by the constant effort to improve the resolution and technical quality of examinations, which will enable increasingly accurate assessment of atherosclerotic plaque composition [67]. Subsequent studies are becoming more precise in determining the effectiveness and seeking optimal sensitivity and specificity of particular imaging techniques [57,67]. Yabushita et al. were the first to show that the accuracy of OCT in diagnosing necrotic lipid plaques was suboptimal [68], Di Vito et al. confirmed these observations and noted the greater precision of a technique combining IVUS and NIRS [69]. Many studies have used the imaging techniques discussed above to evaluate and prove the reversibility of the atherosclerotic process through pharmacological anti-atherosclerotic therapy [70,71,72,73]. Nicholls et al. used IVUS to assess the inhibition of atherosclerosis progression when statins were used as well as a proprotein convertase subtilisin kexin type 9 (PCSK9) inhibitor. The availability of coronary imaging has also allowed studies to identify patients with atherosclerotic plaque erosion amenable to stabilization with antithrombotic therapy without stent implantation [65]. There are an increasing number of reports available regarding the effectiveness of treatment with Inclisiran and monoclonal antibodies against PCSK9. According to the literature, Inclisiran has a good safety profile and can reduce even 50% of the LDL-C level when compared to placebo [74].

Further research is needed to develop the clinical application of imaging studies in diagnosing the status of atherosclerosis, especially within the coronary arteries. Previous reports confirm the sense of intensive therapy focused on reducing risk factors in slowing the progression of the disease. Thanks to imaging studies, it is possible to control not only the volume, but also the composition of atherosclerotic plaques that play a key role in their stabilization, and on which the impact of anti-atherosclerotic therapies still remains to be clarified [67]. Achieving effective therapies to stabilize plaque will better control the increasing prevalence of atherogenic risk factors and reduce the incidence of their cardiovascular complications.

## 5. Conclusions

To summarize, in our manuscript we attempted to overview different methods used to diagnose atherosclerotic plaque. We found literature supporting the usage of an ultrasound examination of plaque but with limitations to shallowly placed vessels, we covered intravascular ultrasound examination and contrast-enhanced with assessments of microbubbles. Furthermore, we described the computed tomography assessment of plaque with their most important patterns: angiography and calcium scoring and with their limitations. We described optimal coherence tomography with its accuracy in assessing the thickness of fibrous cap and limitations in distinctions between calcium and lipids. In the last part, we introduced magnetic resonance imaging and nuclear medicine imaging with its strengths of assessing the density of plaque macrophages and its possibility to predict progression in the calcification score. We also briefly described legal challenges that the introduction of artificial intelligence imaging brings to this field, such as data protection problems and the analysis of different scenarios of diagnostic errors liability. We conclude that both the assessment of quantity and the composition of plaque are important, which can be achieved by further improvement of resolution and quality of examinations.

## Figures and Tables

**Table 1:** The table presents comparison of the different imaging modalities.

Imaging Modality	Advantages	Limitations
USG	Non invasive Enables qualitative assessment of carotid plaques. High spatial resolution required for measuring intima-media thickness.	Limited to shallow vascular beds. Quality is limited by echo windows and calcification. Not indicating whether the thickening is because of intima or media infiltration or hypertrophy. Operator-dependent and has lower reproducibility.
CEUS	Gives information about plaque composition, and structure. Low rate of adverse reactions.	Strongly operator dependent. Cost of contrast media.
IVUS	High image quality direct real-time imaging of atherosclerosis and the vessel. Can assess the structure of atherosclerotic plaque.	Invasive procedure.
CT	Fast. Relatively inexpensive. Suitable for detecting calcifications in atherosclerotic plaque.	Hemorrhage and thrombus could not reliably be distinguished from lipid. Not effective in detecting other components of high-risk plaques: thin-capped fibroma and the presence of inflammatory cells. X-rays patient exposition.
MRI	Not exposing to ionizing radiation. Enables the assessment of intra-plaque hemorrhage and the integrity of the fibrous sheath. Detects positive arterial remodeling in asymptomatic patients with subclinical atherosclerosis.	Prone to unwanted signal loss due to complex flow patterns.

USG—ultrasonography CEUS—contrast-enhanced ultra sonography, CT—computed tomography, MRI—magnetic resonance imaging.

## Data Availability

Not applicable.
